# Identification of key genes associated with heart failure based on bioinformatics analysis and screening of traditional Chinese medicines for the prevention and treatment of heart failure

**DOI:** 10.1097/MD.0000000000035959

**Published:** 2023-12-08

**Authors:** Xu Luo, Rui Wang, Xin Zhang, Xin Wen, Wen Xie

**Affiliations:** a Chengdu University of Traditional Chinese Medicine, Chengdu, Sichuan, China; b Department of Cardiology, Affiliated Hospital of Chengdu University of Traditional Chinese Medicine (Traditional Chinese Medicine Hospital of Sichuan), Chengdu, Sichuan, China.

**Keywords:** bioinformatics analysis, heart failure, key genes, prevention and treatment of traditional Chinese medicine

## Abstract

Heart failure (HF) is the final stage of heart disease. An increasing number of experiments and clinical reports have shown that traditional Chinese medicine (TCM) has many therapeutic effects and advantages in treating HF. In this study, we used bioinformatics methods to screen key genes and predict the components of Chinese herbal medicines with preventive and therapeutic effects on HF. GSE120895 and GSE21610 HF chips were downloaded from the Gene Expression Omnibus database. We screened differentially expressed genes (DEGs). Weighted gene coexpression network analysis was performed to determine key modules. Genes in key modules were used for Gene Ontology and Kyoto Encyclopedia of Genes Genomes analysis to determine the biological functions. Finally, receiver operating characteristic curve analysis was used to screen out key genes, and single-sample GSEA was conducted to screen TCM compounds and effective ingredients of TCM compounds related to HF. We have selected a key module (MeTerquoise) and identified 489 DEGs, of which 357 are up regulated and 132 are down regulated. Gene Ontology and Kyoto Encyclopedia of Genes Genomes analyses indicated that the DEGs were associated with the extracellular matrix, fat metabolism and inflammatory response. We identified IL2, CXCR4, CCL5, THY1, CCN2, and IL7R as key genes. Single-sample GSEA showed that key genes were mainly related to energy metabolism, mitochondrial oxidative phosphorylation, extracellular matrix, and immunity. Finally, a total of 70 TCM compounds and 30 active ingredients of TCM compounds were identified. Bioinformatics methods were applied to preliminarily predict the key genes and TCM compounds involved in HF. These results provide theoretical support for the treatment of HF with TCM compounds and provide targets and research strategies for the development of related new Chinese medicines.

## 1. Introduction

Heart failure (HF) is a clinical syndrome that causes low ventricular pumping and impaired filling function resulting from initial myocardial injury from any source; these effects lead to changes in myocardial structure and function and are the final manifestation of cardiovascular disease and the main cause of death of these patients.^[1]^ HF affects more than 64 million people worldwide.^[2]^ HF is characterized by considerable morbidity and mortality; in recent years, mortality has remained high at ≈50% at 5 years.^[3]^ Importantly, the rapid aging of the population means that the incidence of HF will likely increase further in the coming years. HF is becoming the most important cardiovascular disease in the 21st century.^[4,5]^

Although modern medicine has facilitated significant treatment advancements in improving patient symptoms and reducing mortality rates over the past several decades, the effects are still not ideal.^[6]^ HF patients have a high readmission rate, poor quality of life, and high medical costs, which have become a serious public health problem and a key research issue in clinical medicine^[7]^; these barriers have also burdened the medical system. Overall, modern medical treatment for HF cannot meet clinical needs, and attempts to decrease its social and economic burden have become a major global public health priority.^[2]^

Traditional Chinese medicine (TCM) is the key to unlocking the treasures of Chinese civilization. Traditional Chinese medicines have continued to be a treasure trove, and their compounds play a beneficial role in efforts to treat and cure diseases.^[8,9]^ Over thousands of years of history, traditional Chinese medicine has accumulated rich clinical knowledge and a theoretical basis for treating HF from the perspective of the unity of heaven and humanity, holistic concepts and syndrome differentiation. With the gradual recognition of the effectiveness and safety of traditional Chinese medicine in treating HF. The application of traditional Chinese medicine (TCM) in HF treatment has been gradually accepted. Rational comprehensive treatment with TCM could effectively improve cardiac function and clinical treatment effects and is worthy of clinical application.^[10,11]^ Heart failure is a major health problem worldwide. Traditional Chinese medicine alone or combined with modern medicine strategies can increase the success rate of cardiovascular disease treatment.^[12]^ It is necessary to explore the potential biomarkers and molecular mechanisms related to the occurrence and development of HF to provide more targeted and effective treatment strategies for patients and bring benefits.^[13]^ Bioinformatics is a hybrid science that stores biological information by means of a combination of biological and technical data and plays a massive role in gaining extremely significant information on disorders that is useful in treatment development.^[14]^ Bioinformatics is used in a variety of fields, including genomics, proteomics, and drug discovery.^[15]^ In this present investigation, a microarray data analysis-based gene chip was downloaded for HF and used. Bioinformatics analysis to explore the molecular mechanism and key targets in the progress of HF.^[16,17]^ Traditional Chinese medicine emphasizes the principle of “syndrome differentiation and treatment.” We attempted to screen key targets related to the occurrence and development of HF based on this principle and then selected traditional Chinese medicines and active ingredients that have preventive and therapeutic effects on HF based on these key targets. We hope to provide a theoretical basis for the clinical application of traditional Chinese medicine.

## 2. Materials and methods

### 2.1. Database used and research procedures

**Figure 1.**
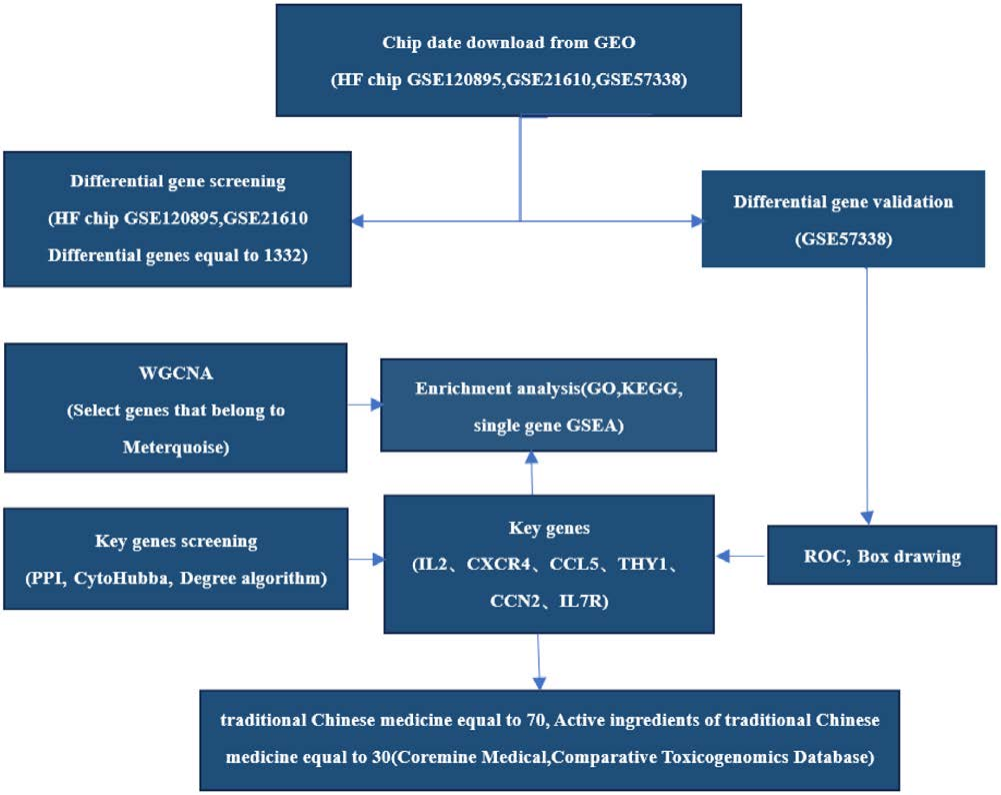
The study procedure.

The basic steps of this study are shown in Figure 1, and the specific databases and software used are shown in Table 1.

**Table 1:** Information about the databases and software used.

Database or software	Website/version	Use
GEO	https://www.ncbi.nlm.nih.gov/geo/	Experimental set, validation set data download
David	https://david.ncifcrf.gov/	GO/KEGG analysis to determine the biological functions
STRING	https://cn.string-db.org	Identifying protein–protein interactions
Cytoscape	Cytoscape_v3.9.1	Key gene screening and visualization, construction of traditional Chinese medicine active ingredients gene target map
GraphPad Prism	GraphPad Prism_v9.5.1	Verify key genes and draw box plots
Coremine Medical	https://www.pubgene.com	Searching for traditional Chinese medicines
CTD	https://ctdbase.org/	Searching for active ingredients in traditional Chinese medicines
R	R_v4.2.3	Screening of differentially expressed genes and data statistical analysis, data mapping
Xiantaoxueshu	https://www.xiantaozi.com/	Obtaining intersection genes and making Venn diagram

### 2.2. Chip data acquisition

The HF mRNA chips GSE120895 and GSE21610 were downloaded from the Gene expression omnibus (GEO) database as the experimental set. The HF chip dataset GSE57338 was downloaded from the GEO database as the validation set.

### 2.3. DEG screening

The differentially expressed genes (DEGs) with significant differences in the HF samples and normal samples on chips GSE120895 and GSE21610 were screened using the conditions of Abs log2Fold Change > 1 and correction *P* < .05 through R 4.2.3 software. Then, we screened the intersecting genes of the 2 chip DEGs by using Xiantao Academic’s Venn diagram. In addition to the screening conditions, the intersecting DEGs also had to have similar upregulation or downregulation of expression before being deleted.

### 2.4. Weighted gene co expression network analysis (WGCNA)

WGCNA was used to identify potential key modules and central genes based on R’s wgcna package. First, all samples were clustered to screen out obvious outliers. Second, a soft threshold power was used to construct a β scale-free network to select the best soft threshold. Third, hierarchical clustering and dynamic tree cutting functions were used to detect modules. Fourth, the correlation between modules and clinical information was analyzed, and key modules were identified.

### 2.5. Gene Ontology (GO) and Kyoto Encyclopedia of Genes Genomes (KEGG) analyses

Key module genes were introduced into DAVID^[18]^ for GO and KEGG analyses to explore the biological functions and signaling pathways. The biological functions of genes can be divided into the following 3 categories: biological processes, cellular components (CC), and molecular functions. We drew bar charts and bubble charts to visualize the enrichment results.

### 2.6. Key gene screening

Genes of key modules were imported into the STRING 11.0 database,^[19]^ and the protein–protein interaction (PPI) network was drawn. PPI network relationships were output in raw data formats and visualized using Cytoscape 3.9.1 software; the cytoHubba plugin was used to screen the top 10 key genes based on the degree algorithm.

### 2.7. Key gene validation

The ROCR package of R was used to evaluate the diagnostic effectiveness of the top ten genes and plot the receiver operating characteristic (ROC) curves. The HF chip GSE57338 dataset was used as the validation set to determine the expression levels of key genes in HF samples and normal samples from the GEO database. Statistical analysis was conducted by GraphPad Prism 9.0.2 software. Nonpaired sample *t*-tests were performed for the expression levels of positive and normal samples, with a difference of *P* < .05 being statistically significant. These results were visualized in the form of a box plot. Finally, the key genes with statistically significant differences in the validation set and an AUC > 0.6 were selected as the important genes of this study; these results suggested that these key genes may be related to HF.

### 2.8. Single-sample GSEA

The ClusterProfiler package of the R package was used to conduct single-sample GSEA. GO enrichment analysis mainly described biological processes, CC, and molecular functions related to genes. KEGG pathway enrichment analysis revealed the related functions and pathways of genes. The correlation coefficients between each gene and all genes in the set were sorted based on the expression value of each gene, and the threshold for enrichment significance was *P* < .05. The enrichment analysis results of the first 10 genes were visualized by drawing a bar chart.

### 2.9. Screening of traditional Chinese medicines and their active ingredients

Key genes were introduced into Coremine Medical to screen traditional Chinese medicine with preventive and therapeutic effects. Key genes were introduced into the comparative toxicogenomics database to screen for active ingredients of traditional Chinese medicines.

### 2.10. Ethics approval and consent to participate

This article belongs to the review category and does not require ethical approval or participants.

## 3. Results

### 3.1. Chip data

The experimental set of the HF chip GSE21610 included 60 HF samples and 8 normal samples. GSE120895 contained 47 dilated cardiomyopathy HF samples and 8 normal samples. The validation set HF chip GSE57338 contained 177 HF samples and 136 normal samples.

### 3.2. Screening of DEGs

**Figure 2.**
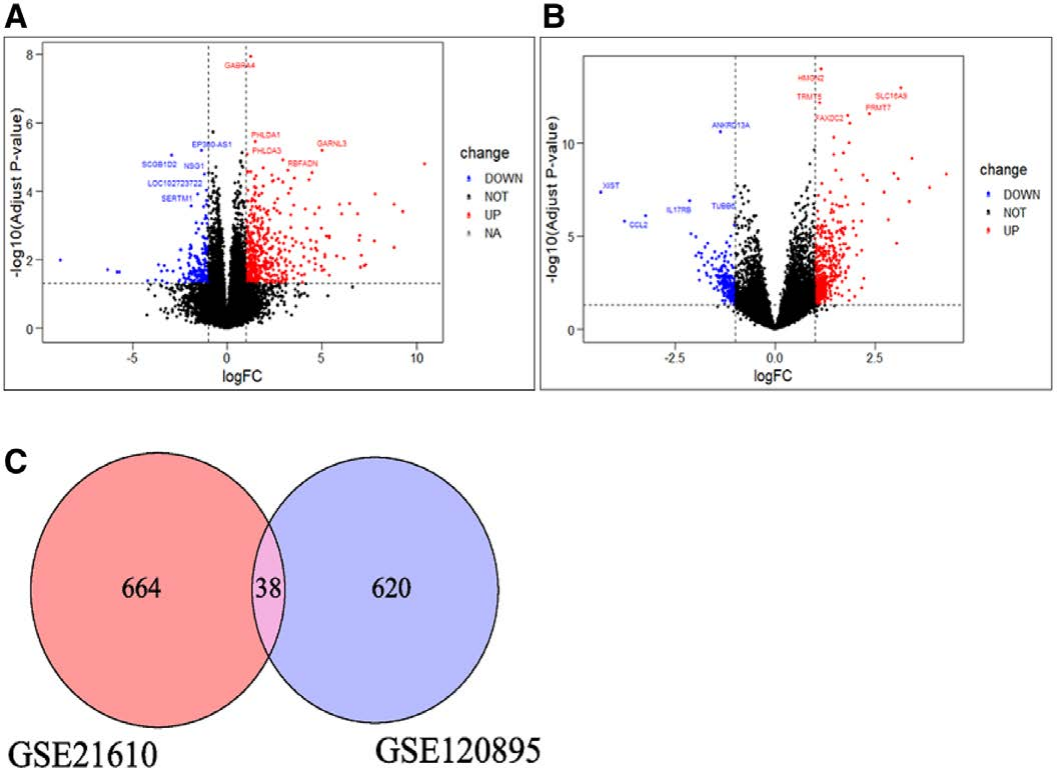
(A) Volcano plot of GSE120895. Blue dots represent downregulated genes, black dots represent nonsignificant genes, gray dots represent genes without data, and red dots represent upregulated genes. The blue genes shown in the figure are the 5 most downregulated genes. The red genes shown in the figure are the 5 genes with the highest degree of upregulation. (B) Volcano plot of GSE21610. Blue dots represent downregulated genes, black dots represent nonsignificant genes, and red dots represent upregulated genes. The blue genes shown in the figure are the 5 most downregulated genes. The red genes shown in the figure are the 5 genes with the highest degree of upregulation. (C) Veen. The intersection of GSE120895 DEGs and GSE21610 DEGs were identified as identical genes that could be eliminated. DEGs = differentially expressed genes.

Using the GSE21610 chip, we identified 702 HF DEGs through the comparative analysis of HF samples and normal samples, which included 456 upregulated genes and 246 downregulated genes. Using the GSE120895 chip, we identified 658 HF DEGs, which included 487 upregulated genes and 171 downregulated genes. These results are shown in Figure 2A and B. Thirty-eight intersecting genes were obtained by using the Venn diagram plot (Fig. 2C), and 1332 DEGs that included 924 upregulated genes and 408 downregulated genes were ultimately identified by deleting identical genes with similar increases or decreases in expression.

### 3.3. WGCNA results

**Figure 3.**
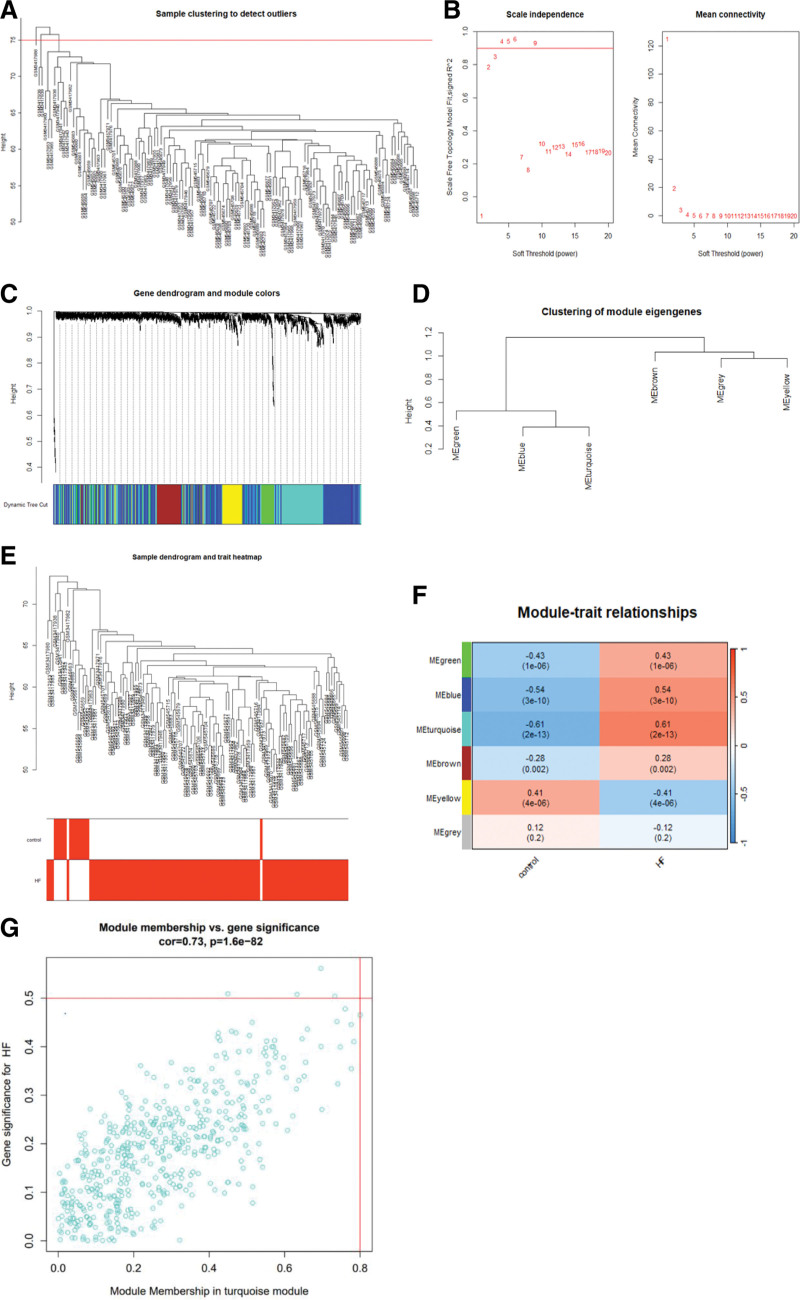
Weighted gene co-expression network analysis based on DEGs. (A) Sample cluster cut graph. The cutting height was set to 75, and unsuitable samples were removed. (B) Fitting coefficient R2 as a function of a soft threshold parameter in the scale-free topology model and mean connectivity as a function of a soft threshold parameter. (C) Cluster dendrogram. Genes are divided into various modules by hierarchical clustering, and different colors represent different modules, among which gray defaults to genes that cannot be classified into any module. (D) Module eigengene dendrogram. (E) Clustering dendrogram of the clinical traits and data from 120 HF samples. Red represents “HF,” and white represents “control” for the variable. (F) The heatmap of modules and heart failure correlation. In the figure, red represents a positive correlation, blue represents a negative correlation, and the darker the color is, the stronger the correlation. Each frame represents the correlation coefficient, with the corresponding *P* value in brackets. (G) Scatter plots showing the correlation between module membership (*X*-axis) and gene significance (*Y*-axis) in the turquoise module. DEGs = differentially expressed genes, HF = heart failure.

The samples from the GSE120895 dataset and the GSE21610 dataset were merged, resulting in a total of 123 samples. R’s wgcna package was used to perform WGCNA on normal and HF samples. After checking for missing values, 1294 differentially expressed genes were obtained. The cutting line height was set to 75, and 3 samples that did not meet the height were deleted (Fig. 3A). An optimal soft threshold of 3 was selected based on the scale-free topology standard R^2^ equal to 0.9. (Fig. 3B). At least 50 genes were set in the gene module tree, and a total of 6 modules were obtained, namely, Megreen, Meblue, Meterquoise, Mebrown, Meyellow, and Gray (Fig. 3C). The results of hierarchical clustering, adjacency relationship and the heat graph, bubble chart (Fig. 3D–G) showed that the relationship between Meterquoise and HF was relatively close. The Meterquoise module had a total of 489 genes, of which 357 are up regulated and 132 are down regulated. Next, the MeterQuoise module genes were subjected to functional enrichment analysis and central gene analysis.

### 3.4. GO and KEGG analysis results

**Figure 4.**
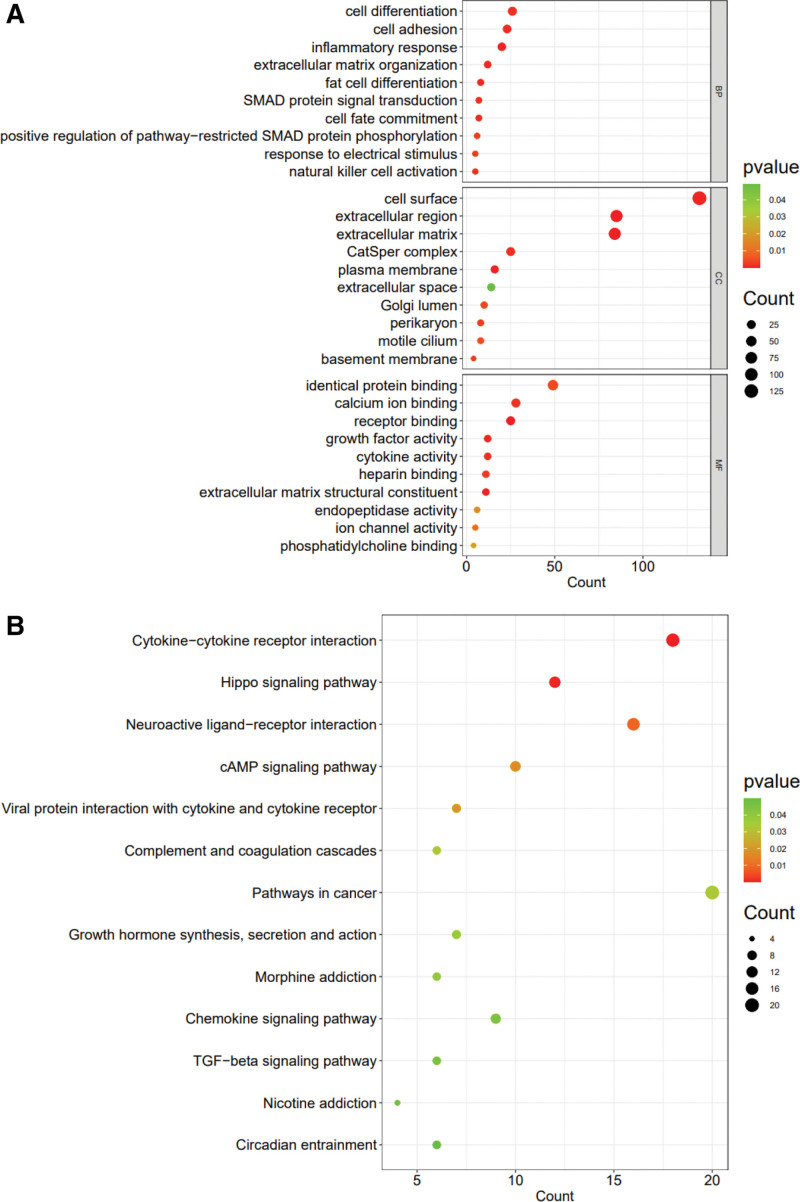
The coenriched GO entries and KEGG pathways. (A) GO analysis of the functional enrichment of the turquoise module. The dot size reflects the number of enriched genes, and the color indicates the significance of enrichment. The BP enrichment results, CC enrichment results, and MF enrichment results are represented from top to bottom. (B) KEGG pathways of the turquoise module. The dot size reflects the number of enriched genes, and the color indicates the significance of enrichment. BP = Biological processes, CC = cellular components, GO = Gene Ontology, KEGG = Kyoto Encyclopedia of Genes and Genomes, MF = molecular functions.

Meterquoise module genes were introduced into DAVID for GO analysis and KEGG pathway analysis. GO analysis (Fig. 4A) showed that DEGs were mainly enriched in extracellular matrix (ECM) organization and structural constituent, inflammatory response, fate cell differentiation, intracellular signal transduction, cell surface receptor signal transduction, growth factor activity, response to electrical stimulus, cell adhesion, etc. KEGG analysis (Fig. 4B) showed that genes were significantly enriched in cytokines and cytokine receptors, the Hippo signaling pathway, neuroactive ligand–receptor interaction and the cAMP signaling pathway. These enrichment analysis results may be closely related to HF.

### 3.5. Key gene screening results

**Figure 5.**
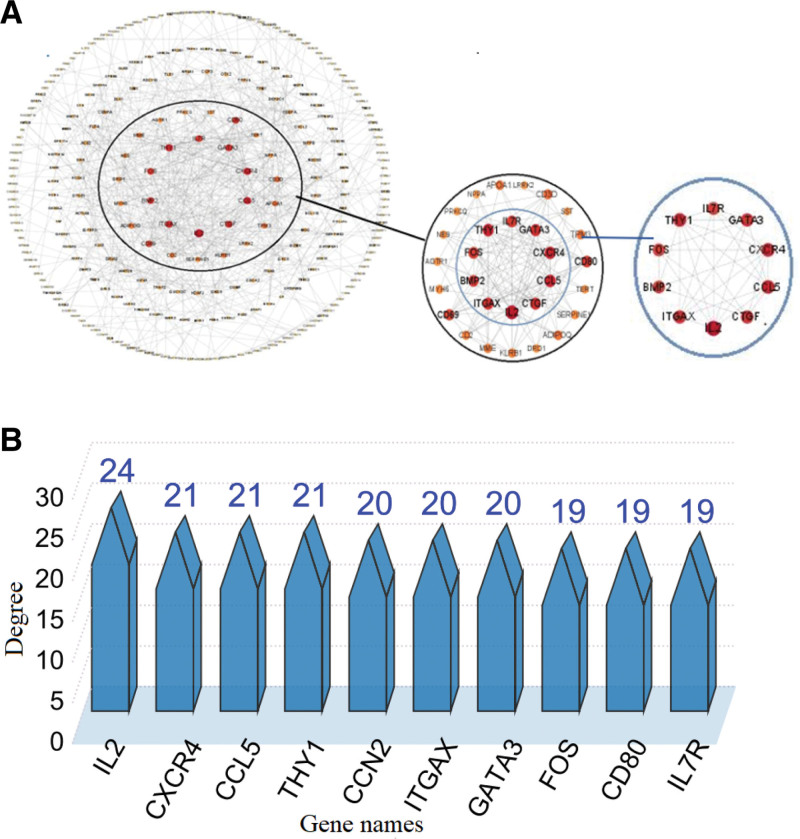
(A) Top 10 hub genes of the HF network. (B) Histogram of genes and degrees. HF = heart failure.

Meterquoise module genes were imported into the STRING 11.5 database to generate a list of PPI relationships in data format. Cytoscape 3.9.1 software was used to visualize PPI data and gave 270 genes and 606 edges (Fig. 5A). Then, the degree algorithm of the cytoHubba plugin was used to screen the top 10 key genes (Fig. 5B), including interleukin-2 (IL2), C-X-C chemokine receptor type 4 (CXCR4), C-C motif chemokine 5 (CCL5), Thy-1 membrane glycoprotein (THY1), CCN2, ITGAX, GATA3, FOS, CD80, and interleukin-7 receptor (IL7R). Among them, 8 upregulated genes are involved in the regulation of immune and inflammatory response, signal transduction, cell growth, and differentiation. Two downregulated genes mainly regulate the inflammatory response and activate inflammatory cells (Table 2).

**Table 2:** Top 10 hub genes from the HF information sheet.

Sequence number	Gene name	Degree value	Changes encoding	Protein function annotation
1	IL2	24	UP	Produced by T cells in response to antigenic or mitogenic stimulation, this protein is required for T-cell proliferation and other activities crucial to regulation of the immune response
2	CXCR4	21	UP	Chemokine receptor, that transduces a signal by increasing intracellular calcium ion levels and enhancing MAPK1/MAPK3 activation.
3	CCL5	21	UP	Chemokine 5; Chemoattractant for blood monocytes, memory T-helper cells and eosinophils
4	THY1	21	UP	May play a role in cell–cell or cell-ligand interactions during synaptogenesis and other events in the brain
5	CTGF (CCN2)	20	UP	Connective tissue growth factor. Major connective tissue mitoattractant secreted by vascular endothelial cells. Promotes proliferation and differentiation of chondrocytes.
6	ITGAX	20	DOWN	It mediates cell–cell interaction during inflammatory responses
7	GATA3	20	UP	Transcriptional activator which binds to the enhancer of the T-cell receptor alpha and delta genes. Required for the T-helper 2 (Th2) differentiation process following immune and inflammatory responses;
8	FOS	19	UP	Signal transduction, cell proliferation and different
9	CD80	19	DOWN	Involved in the costimulatory signal essential for T- lymphocyte activation.
10	IL7R	19	UP	Implicated in the development of the hematopoietic system, participate in cell growth, development, or differentiation

### 3.6. Key gene verification

**Figure 6.**
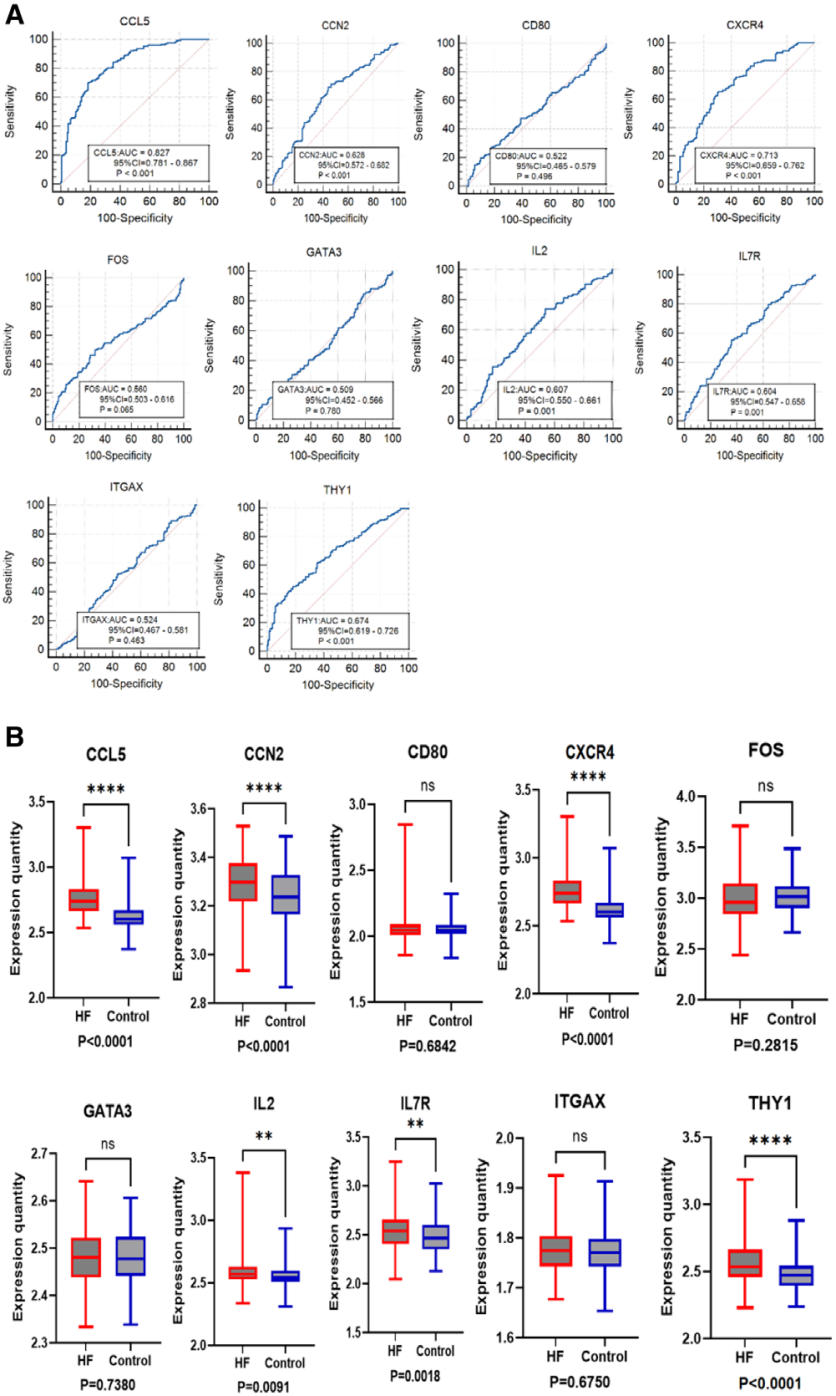
(A) ROC diagnostic curves of key genes in GSE57338 data set. (B) Expression difference of the top 10 DEGs in the HF validation set: ****P < .0001,***P < .001, **P < .01,*P < .05. DEGs = differentially expressed genes, HF = heart failure, ROC = receiver operating characteristic.

We further evaluated the diagnostic value of IL2, CXCR4, CCL5, THY1, CCN2, ITGAX, GATA3, FOS, CD80, and IL7R and used ROC curves to validate the diagnostic value of candidate biomarkers (Fig. 6A). Among them, IL2, CXCR4, CCL5, THY1, CCN2, and IL7R had high accuracy with AUC > 0.6. We used the HF chip GSE57338 dataset as the validation set and determined the expression levels of key genes in HF samples and normal samples from the GEO database. Statistical analysis was conducted by GraphPad Prism 9.0.2 software. Nonpaired sample t tests were performed for the expression levels of HF samples and normal samples, and *P* < .05 was considered statistically significant. The results were visualized in the form of a box plot (Fig. 6B). Finally, the statistically significant key genes in the validation set and the key genes identified in the ROC curve were the primary results of this study. Six key genes were identified, namely, IL2, THY1, CXCR4, IL7R, CCL5, and CCN2, which may be related to HF.

### 3.7. Single-sample GSEA results

**Figure 7.**
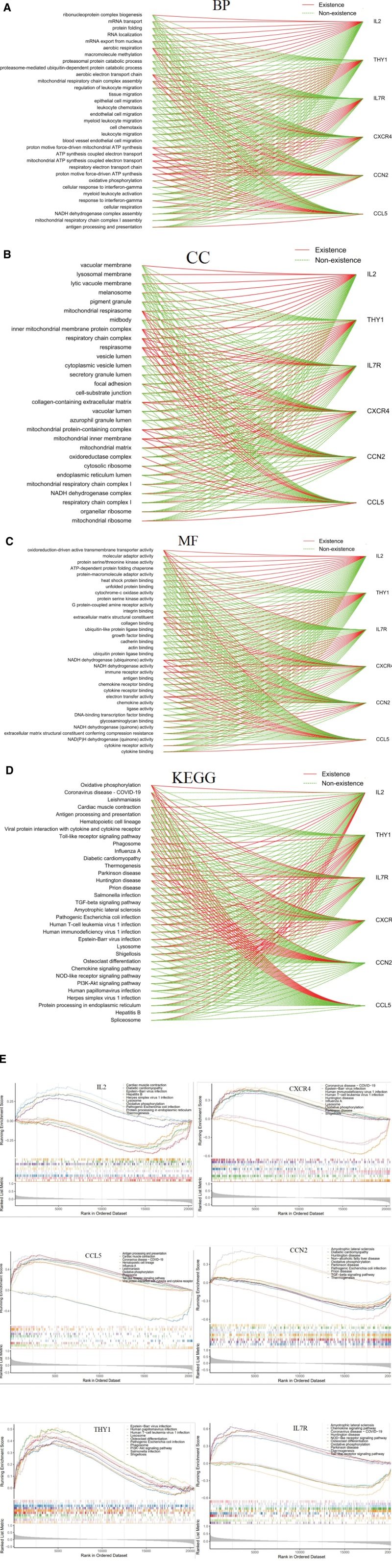
The coenriched GO entries and KEGG pathways of the 6 key genes based on GSEA. (A) Coenriched GO terms in the BP category. (B) Coenriched GO terms in the CC category. (C) Coenriched GO terms in the MF category. (D) Coenriched KEGG pathways. The solid red line represents the results of genes enrichment, while the dashed green line represents the results of genes non enrichment. (E) Specific KEGG pathways of GSEA for 6 key genes. BP = Biological processes, CC = cellular components, GO = Gene Ontology, KEGG = Kyoto Encyclopedia of Genes and Genomes, MF = molecular functions.

We conducted GSEA on a single biomarker based on GO and KEGG gene sets to explore the potential related functions and pathways in HF. The top 10 items in the GO (biological processes), GO (CC), and GO (molecular functions) categories of key genes are shown in Figure 7A–C. Biological processes, IL2, CXCR4, IL7R, CCL5, and CCN2 are involved in the aerobic electron transport chain. CXCR4, IL7R, CCL5, and CCN2 are involved in proton motive force-driven mitochondrial adenosine triphosphate (ATP) synthesis, ATP synthesis coupled electron transport, mitochondrial ATP synthesis coupled electron transport, respiratory electron transport chain, and proton motive force-driven ATP synthesis. IL2, CXCR4, CCL5, and CCN2 are involved in aerobic respiration. CXCR4, THY1, and CCN2 are involved in mitochondrial respiratory chain complex assembly. IL7R, CCL5, and CCN2 are involved in oxidative phosphorylation. For CC, all 6 genes are involved in the inner mitochondrial membrane protein complex. IL2, CXCR4, IL7R, CCL5, and CCN2 are involved in the mitochondrial respirasome, respiratory chain complex, and respirasome. CXCR4, IL7R, CCN2, and THY1 are involved in collagen-containing ECM. THY1, CXCR4, IL7R, CCL5, and CCN2 are involved in the mitochondrial protein-containing complex. CXCR4, IL7R, CCL5, and CCN2 are involved in the mitochondrial inner membrane. For molecular functions, IL2, CXCR4, IL7R, CCL5, and CCN2 were involved in oxidoreduction-driven active transmembrane transporter activity. THY1, CXCR4, and CCN2 are involved in integrin binding. THY1, CXCR4, CCN2, and CCL5 are involved in ECM structural constituents. THY1, IL7R, CXCR4, CCN2, and CCL5 are involved in NADH dehydrogenase (ubiquinone) activity and NADH dehydrogenase activity. CXCR4, IL7R, and CCL5 are involved in immune receptor activity. IL7R, CXCR4, CCN2, and CCL5 are involved in electron transfer activity. Based on KEGG analysis, IL7R, CXCR4, CCN2, CCL5, and IL2 were involved in oxidative phosphorylation (Fig. 7D and E).

### 3.8. Results of the screening of traditional Chinese medicines and active ingredients

Six key genes related to HF, including IL2, CXCR4, CCL5, THY1, CCN2, and IL7R, were used as predictive targets. In the Coremine Medical Database, Chinese herbal.

**Figure 8.**
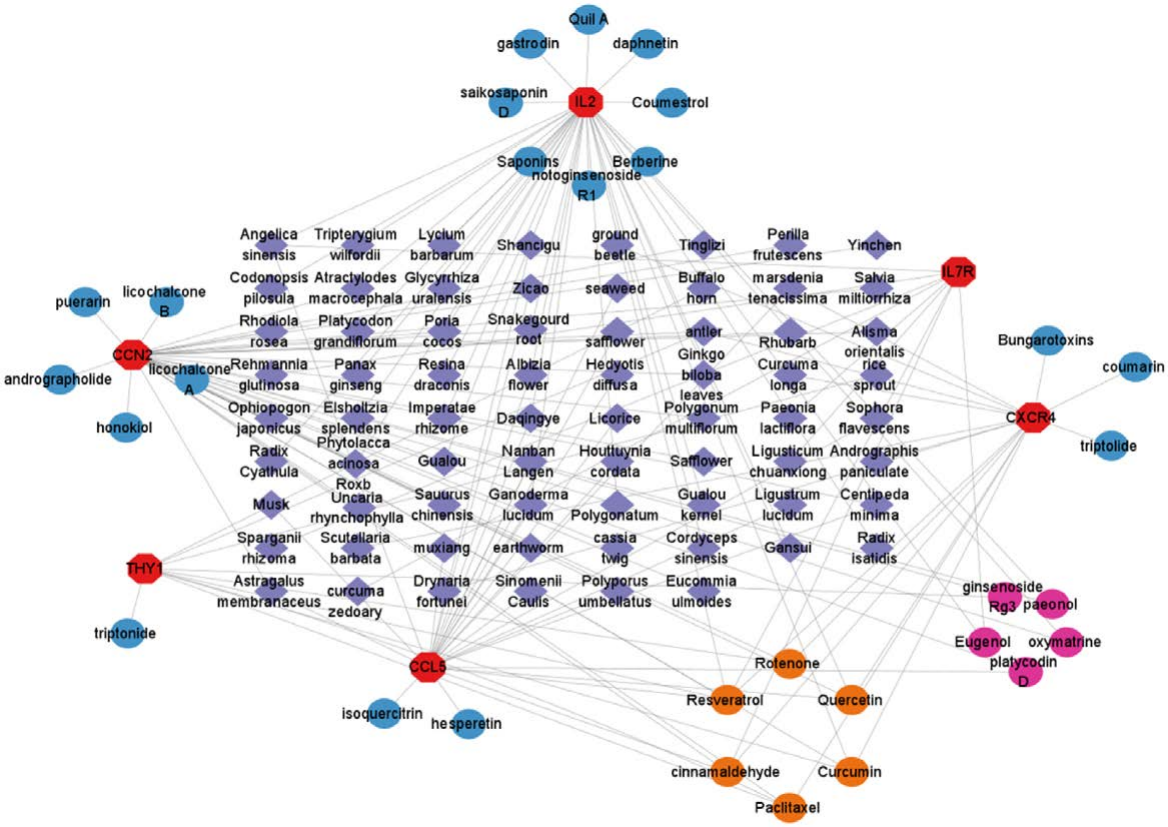
Interaction network diagram of traditional Chinese medicine active components that target the core genes. The red octagon represents the key gene and the purple diamond represents the Chinese medicine acting on the key gene; the blue circle, the orange circle and the purple red circle are the active ingredients of Chinese medicines that act on the genes, the blue circle represents ingredients that only act on one gene, the purple red circle represents ingredients that act on 2 genes, and the orange circle represents ingredients that act on 3 or more genes. HF = heart failure.

Medicines with biological effects on each predicted target were selected based on *P* < .05, and the drug was included in the *Pharmacopoeia of the People’s Republic of China*. The IL7R gene was not found in the Coremine medical database for corresponding Chinese herbal medicines according to the screening criteria of *P* < .05. Therefore, we identified traditional Chinese medicines that act on the IL7R gene through a literature review,^[20]^ as shown in Table 3. These traditional Chinese medicines can increase the expression of Bcl-2 and reduce the expression of Bax, which may be related to the role of IL7R in promoting cell survival by regulating antiapoptotic and proapoptotic factors.^[21]^ The frequency was statistically analyzed, and a total of 70 traditional Chinese medicines targeted the 6 gene targets. There were 17 types of traditional Chinese medicines that targeted key genes at least twice, including *Radix isatidis* (banlangen), Daqingye, *Ganoderma lucidum*, Licorice, *Centipeda minima*, Albizia flower, *Marsdenia tenacissima*, safflower, *Angelica sinensis, Polyporus umbellatus*, antler, Polygonatum, *Centipeda minima*, Snakegourd root, *Poria cocos, Atractylodes macrocephala*, and Gualou. We used the comparative toxicogenomics database database to analyze the 6 key genes and screen potential active ingredients of traditional Chinese medicines for the treatment of HF, and a total of 30 types of therapeutic traditional Chinese medicine ingredients were obtained. The active ingredients of traditional Chinese medicine that target more than 3 key genes include resveratrol, quercetin, rotenone, cinnamaldehyde, paclitaxel, and curcumin. These results are shown in Table 4. Finally, we constructed an interaction network of therapeutic traditional Chinese medicine active ingredients that target core genes by using Cytoscape 3.9.1 software, as shown in Figure 8.

**Table 3:** Traditional Chinese medicines that target the 6 key genes.

Gene	Name of traditional Chinese medicine
IL2	*Radix Cyathula, Ganoderma lucidum, Cordyceps sinensis, Angelica sinensis, Codonopsis pilosula, Platycodon grandiflorum, Panax ginseng, Elsholtzia splendens, Phytolacca acinosa* Roxb, Polygonatum, Gansui, *Tripterygium wilfordii, Atractylodes macrocephala, Poria cocos, Resina draconis, Imperatae rhizome*, Gualou, *Gualou kernel, Radix isatidis* (banlangen), *Lycium barbarum, Glycyrrhiza uralensis*, Snakegourd root, Albizia flower, Daqingye
CXCR4	*Andrographis paniculate, Scutellaria barbata, Drynaria fortunei*, ground beetle, marsdenia tenacissima, antler, Turmeric (*Curcuma longa*)
CCL5	Nanban Langen, *Ligustrum lucidum, Astragalus membranaceus*, Shancigu (*Rhizoma Pleionis*), Zicao, *Radix isatidis* (banlangen), Daqingye, *Ganoderma lucidum*, Licorice, *Houttuynia cordata, Centipeda minima*, Albizia flower, Sinomenii Caulis, marsdenia tenacissima, seaweed, safflower, *Hedyotis diffusa, Angelica sinensis, Sophora flavescens*, Musk, muxiang (Radix Aucklandiae), *Polyporus umbellatus*
THY1	Buffalo horn, antler, Polygonatum, rice sprout
CCN2	*Centipeda minima, Uncaria rhynchophylla,* earthworm, *Eucommia ulmoides*, Tinglizi (Semen Lepidii Apetali), *Salvia miltiorrhiza*, Snakegourd root, *Ginkgo biloba* leaves, *Polygonum multiflorum, Poria cocos, Sauurus chinensis*, cassia twig, Rhubarb, *Perilla frutescens, Rhodiola rosea, Alisma orientalis, Ophiopogon japonicus, Atractylodes macrocephala*, Safflower, *Cordyceps sinensis, Polyporus umbellatus*, antler, *Astragalus membranaceus*, Yinchen, Gualou
IL7R	*Rehmannia glutinosa, Angelica sinensis, Paeonia lactiflora, Ligusticum chuanxiong, Sparganii rhizoma*, curcuma zedoary

**Table 4:** Active ingredients of traditional Chinese medicine targeting 6 key genes.

Gene	Active ingredient	Compound number	Gene	Active ingredient	Compound number
IL2	Notoginsenoside R1	C072936	THY1	Curcumin	D003474
	Oxymatrine	C037573		Ginsenoside Rg3	C097367
	Paeonol	C013638		Paclitaxel	D017239
	Quercetin	D011794		Resveratrol	D000077185
	Quil A	C046386		Rotenone	D012402
	Resveratrol	D000077185		Triptonide	C001899
	Saikosaponin D	C025759	CCN2	Andrographolide	C030419
	Saponins	D012503		Rotenone	D012402
	Berberine	D001599		Cinnamaldehyde	C012843
	Coumestrol	D003375		Curcumin	D003474
	Curcumin	D003474		Ginsenoside Rg3	C097367
	Daphnetin	C039952		Honokiol	C005499
	Eugenol	D005054		Licochalcone A	C070840
	Gastrodin	C045345		Licochalcone B	C541528
CXCR4	Bungarotoxins	D002038		Oxymatrine	C037573
	Cinnamaldehyde	C012843		Paclitaxel	D017239
	Coumarin	C030123		Paeonol	C013638
	Curcumin	D003474		Platycodin D	C108953
	Paclitaxel	D017239		Puerarin	C033607
	Quercetin	D011794		Quercetin	D011794
	Resveratrol	D000077185		Resveratrol	D000077185
	Rotenone	D012402	IL7R	Cinnamaldehyde	C012843
	Triptolide	C001899		Resveratrol	D000077185
CCL5	Cinnamaldehyde	C012843		Eugenol	D005054
	Hesperetin	C013015			
	Isoquercitrin	C016527			
	Platycodin D	C108953			
	Quercetin	D011794			
	Resveratrol	D000077185			

## 4. Discussion

### 4.1. Key gene analysis

We conducted bioinformatics analysis on an independent cohort of HF patient samples and screened the Meterquoise modules most related to HF through weighted gene coexpression network analysis. We conducted GO and KEGG analyses on the MeterQuoise module genes, and GO functional enrichment showed that the biological processes of DEGs between HF patients and normal individuals were mainly involved in the inflammatory response, cell adhesion, ECM organization, fat cell differentiation, etc. The CC were mainly enriched in extracellular space, extracellular region, ECM, plasma membrane, etc. The enriched molecular functions were closely related to receptor binding, ECM structural components, and growth factor activity. KEGG results showed that the specific DEGs were mainly involved in pathogenesis pathways such as the cytokine–cytokine receptor interaction, the Hippo signaling pathway, and the cAMP signaling pathway. Inflammatory reactions promote myocardial injury, fibrosis remodeling, and dysfunction.^[22]^ Cardiac fibrosis, which is characterized by excessive deposition of ECM proteins in the myocardium, distorts the structure of the myocardium and promotes the progression of arrhythmias and cardiac dysfunction.^[23]^ Hippo signaling regulation leads to a response to the failure phenotype, including abnormal myocardial cell growth and death, indicating that this pathway plays a role in chronic stress.^[24]^ Hippo signal activation promotes the development of dilated cardiomyopathy by inhibiting gene-mediated mitochondrial damage. This Hippo mitochondrial damage pathway may have clinical significance for HF, as mitochondrial metabolism is significantly impaired in patients diagnosed with HF; this is one of the reasons for myocardial cell dysfunction.^[25]^ Cyclic adenosine monophosphate is the primary second messenger for many organs. Especially in the cardiovascular system, it regulates many processes in the cardiovascular system, from the short-term effects of contraction/relaxation to the long-term effects of cell growth/survival. For example, cyclic adenosine monophosphate enhancement, especially through β adrenergic receptor stimulation, has crucial positive inotropic and myotonic effects when cardiac demand increases. It can also regulate calcium and many other physiological processes, including homeostasis, pulse rate, myocardial contractility and cell death.^[26,27]^ Single-sample GSEA was used to enrich the potential related functions and pathways of key genes. The GSEA results showed that these genes were related to mitochondrial metabolism and mitochondrial oxidative phosphorylation, and a large amount of energy was needed to promote the function and activity of the heart through oxidative phosphorylation in mitochondria. The oxidative phosphorylation of mitochondria is strictly regulated by the renewal of ATP, which provides fuel for cardiac contraction and relaxation. In HF, changes in myocardial redox regulation occur, and this mechanical energy coupling is disrupted, leading to bioenergy mismatch and the production of reactive oxygen species.^[28]^ Changes in cardiac energy metabolism and mechanical energy coupling have been identified in almost all stages and causes of HF.^[29]^ In conclusion, these results showed that inflammation, metabolism, oxidative stress, ECM remodeling, an imbalance between the formation and breakdown of ECM and mitochondrial oxidative phosphorylation are closely related to the occurrence and development of HF.

Six key genes were divided into 2 categories. The first category was those involved in signal transduction and ECM remodeling, which included THY1 and CCN2. THY-1 is expressed in fibroblasts, myofibroblasts, thymocytes, mesenchymal stem cells, cancer stem cells, ovarian cancer cells, endothelial cells, neurons, neuronal smooth muscle cells, hematopoietic cells, and plant cells.^[30,31]^ THY1 interacts with other molecules and may function as part of a multimolecular complex, affecting several important intracellular signaling cascades.^[32]^ CCN2 is known as a connective tissue growth factor, exists in the tissue structure and regulates cell proliferation, survival, differentiation, adhesion, migration and ECM production. CCN2-mediated increases in ECM synthesis play a role in a variety of fibrotic diseases.^[33]^ THY-1 regulates changes in the cell phenotype in response to external stimuli, regulates basic characteristics, such as pluripotency, differentiation and survival, and plays an important role in cell differentiation and regeneration.^[31]^ The regulation of THY1 and CCN2 expression plays an important role in the pathogenesis of heart disease. THY1 inhibits the proliferation of dermal fibroblasts and promotes their apoptosis and differentiation. If abnormal regulation occurs, myocardial fibrosis and remodeling can be observed.^[34]^ Li Y et al found that mice with complete knockout of the THY1 gene showed more severe cardiac dysfunction and fibrosis,^[35]^ and CCN2/CTGF (connective tissue growth factor) was strongly induced in HF.^[36]^ Dorn L E et al found that CCN2 secreted by myocardial cells is not profibrotic, while CCN2 derived from fibroblasts can regulate cardiac fibrosis.^[37]^ The second category is those involved in inflammatory responses, such as IL2, IL7R, CCL5, and CXCR4. IL2 and IL7R belong to the interleukin family, and interleukins and their receptors play an important role in antitumor immune responses or are also related to cardiac immunity. Blum A et al believed that interleukin-2 is associated with complex syndromes of heart depression and HF.^[38]^ Wang H et al believed that interleukin-2 can activate regulatory T cells and alleviate the progression of HF.^[39]^ CCL5 and CXCR4 are chemokine families, and animal experiments have shown that CCL5 activates immune responses and leads to significant upregulation of many inflammatory factors, promoting myocardial cell hypertrophy, cardiac remodeling, and interstitial fibrosis, thereby causing myocardial damage.^[40]^ Meanwhile, the chemokine CXCR4 can cause myocardial remodeling, reduce scar volume, and prevent ventricular remodeling and the progression of HF.^[41]^ Simultaneously, endothelial differentiation leads to the formation of new blood vessels in the graft, allowing for the maintenance of capillary density and function.^[42]^

### 4.2. Prediction and analysis of traditional Chinese medicines and active ingredients

**Figure 9.**
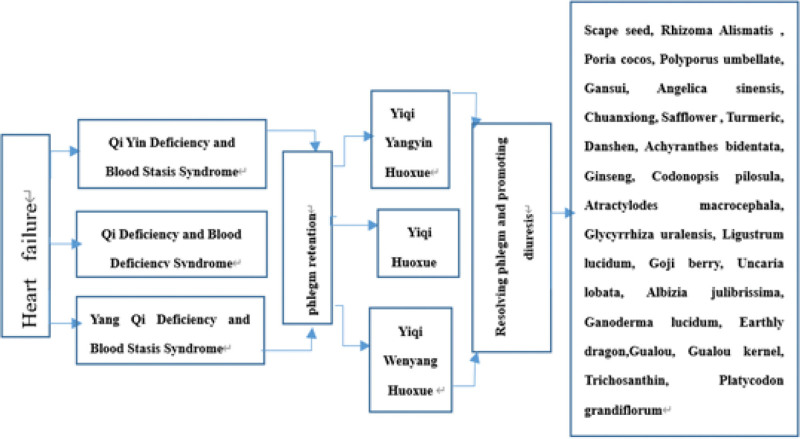
Dialectical treatment and treatment of HF with traditional Chinese medicine.

The 70 traditional Chinese medicines predicted to be related to key genes in the treatment of HF can be divided into the following types of treatments: (1) Promotion of diuresis and removal of dampness. This treatment has the effect of reducing swelling and clearing gonorrhea by clearing heat and promoting diuresis and dampness. These medicines include Medical Tinglizi (Semen Lepidii Apetali), *Alisma orientalis, Poria cocos, Polyporus umbellatus, Phytolacca acinosa* Roxb, *Tripterygium wilfordii*, Sinomenii Caulis, Yinchen, *Sophora flavescens*, Gansui, *Sauurus chinensis*, seaweed, *Imperatae rhizome*, and *Elsholtzia splendens*. (2) Clearing heat, detoxifying and cooling blood. This treatment has the functions of cooling and detoxifying, dispelling dampness and heat pathogenic factors and is medicinally part of the mechanisms of *Andrographis paniculate, Scutellaria barbata*, Nanban Langen, Shancigu (*Rhizoma pleionis*), *Radix isatidis* (banlangen), Daqingye, *Houttuynia cordata, Centipeda minima, Hedyotis diffusa*, Zicao, Buffalo horn, etc. (3) Regulating blood circulation, promoting blood circulation, and removing blood stasis. These treatments can promote blood circulation and remove blood stasis. Medicinal samples include *Angelica sinensis, Ligusticum chuanxiong*, safflower, turmeric (*Curcuma longa*), *Salvia miltiorrhiza*, Radix Cyathula, *Resina draconis, Sparganii rhizoma, Drynaria fortunei, Ginkgo biloba* leaves, and curcuma zedoary. (4) Nourishing qi and blood. With a sweet taste, these medicines can nourish vitality, promote fluid production and nourish blood. They include ginseng, *Codonopsis pilosula, Atractylodes macrocephala*, liquorice, *Ligustrum lucidum, Lycium barbarum, Polygonum multiflorum, Cordyceps sinensis, Astragalus membranaceus, Astragalus membranaceus, Rhodiola rosea, Rehmannia glutinosa*, Polygonatum, muxiang (Radix Aucklandiae) and other medicines. (5) Nourishing and calming the mind. These are medicinal herbs used to nourish the heart, blood, and vital energy and calm the mind. Traditional Chinese medicines include Albizia flowers, Ganoderma lucidum, and earthworms. (6) Ventilating the lung and resolving phlegm. These medicines have the function of reducing fever, resolving phlegm, broadening the chest and dispersing nodules. Medicinally used forms include Gualou, Gualou kernel, Snakegourd root, and Platycodon grandiflorum. HF can be classified into the categories of “asthma syndrome,” “heart water,” “palpitations,” “phlegm” and “yin” in traditional Chinese medicine. *The consensus of Traditional Chinese Medicine Diagnosis and Treatment Experts on Chronic Heart Failure*^[43]^ is that the traditional Chinese medicine syndrome types of HF can be summarized into the following 3 basic syndrome types: qi deficiency and blood stasis, qi yin deficiency and blood stasis, and yang qi deficiency and blood stasis. However, phlegm retention can be seen among all syndrome types. In this study, selected drugs closely related to the differentiation and treatment of HF aligned with the principles of traditional Chinese medicine differentiation and treatment, as shown in Figure 9.

Ventricular remodeling is the pathological and physiological basis for the occurrence and development of HF. Myocarditis, myocardial infarction, and excessive hemodynamic load are the initiating factors of HF, while decreased cardiac function leads to hemodynamic disorders, inducing neuroendocrine activation, oxidative stress mechanisms and cytokine release leading to inflammatory reactions, as its subsequent factors.^[44]^ All of these mechanisms eventually lead to a vicious cycle of worsening HF.^[45]^ Qi-tonifying drugs, heart-nourishing drugs and sedative drugs can effectively inhibit the excessive activation of the neuroendocrine systems. Blood-activating and meridian-unblocking drugs and diuretic and swelling-reducing drugs can improve the blood supply to the heart, eliminate stagnant blood and phlegm in the heart meridians, increase heart contractions and effectively inhibit ventricular remodeling^[46]^ to inhibit the occurrence and development of HF. Ginseng, *Astragalus membranaceus*, and *Codonopsis pilosula* are known to tonify qi and nourish the heart. These factors have been proven to improve the abnormal expression of enzymes related to cardiac energy metabolism and cardiac fatty acid oxidation, protect the integrity of mitochondrial membranes, exert antiapoptotic effects and inhibit cardiac inflammation and fibrosis.^[47–50]^ The sedative drugs represented by *Uncaria rhynchophylla* can significantly reverse cardiac remodeling, improve cardiac structure and function, inhibit oxidative stress and inflammation, and resist thrombosis.^[51]^ The blood activating and stasis resolving drugs represented by *Salvia miltiorrhiza* and *Ligusticum chuanxiong* can alleviate the contraction of the aorta caused by vasoconstrictor substances, inhibit tumor necrosis factor expression and the activation of nuclear transcription factors induced by it, and improve the inflammatory damage related to HF. The total phenolic acids of *Salvia miltiorrhiza* can clear oxygen free radicals, inhibit reoxidation, prevent calcium ion influx, and alleviate myocardial damage.^[52,53]^ These medicines can improve the symptoms of myocardial ischemia and improve microcirculation dysfunction and organ damage caused by ischemia–reperfusion (I/R).^[54]^ Diuretic and anti-inflammatory drugs represented by Tinglizi (Semen Lepidii Apetali) and *Alisma orientalis* can improve myocardial ischemia–reperfusion injury, protect myocardial cells, and inhibit ventricular remodeling.^[55,56]^ Thirty active ingredients of traditional Chinese medicine that target key genes were identified. The active ingredients of traditional Chinese medicine that target 3 or more key genes include resveratrol, quercetin, rotenone, cinnamaldehyde, paclitaxel, and curcumin. These active ingredients can have an impact on the occurrence and development of HF. Sung M M et al demonstrated that resveratrol can alleviate the severity of the mouse HF phenotype by reducing cardiac fibrosis, improving molecular and structural remodeling of the heart, and enhancing diastolic function, vascular function, and energy metabolism.^[57]^ Quercetin has significant cardiac benefits based on inhibiting LDL oxidation and nonendothelial-dependent vasodilation, reducing adhesion molecules and other inflammatory markers and protecting nitric oxide and endothelial function under oxidative stress conditions.^[58]^ Traditional Chinese medicines exert cardioprotective effects in a multicomponent and multitarget manner. Active ingredients of traditional Chinese medicines can also improve cardiac function through multiple targets and pathways. Using traditional Chinese medicine, clinicians can choose different treatment methods and corresponding traditional Chinese medicines for HF according to the different syndromes of patients, thus leading to personalized and differentiated treatment plans. Compared with Western medicine, traditional Chinese medicine has potential advantages in treating chronic HF, such as fewer side effects, a lower incidence of adverse reactions, reduced occurrence and development of HF, and improved patient prognosis.

## 5. Conclusion

In summary, in this study, we applied bioinformatics analysis to preliminarily predict potential targets closely related to HF and reveal their biological processes. In addition, traditional Chinese medicines with preventive and therapeutic effects on HF were selected based on key targets. These relevant results need to be verified through subsequent experiments and clinical studies to provide a reference for the development of new Chinese medicines.

## Acknowledgments

Thank the Gene Expression Omnibus database for the datasets GSE120895/GSE21610/GSE57338. Thank the funding of Key Project of Sichuan Provincial Department of Science and Technology (No. 2022YFS0395).

## Author contributions

**Conceptualization:** Wen Xie.

**Data curation:** Xu Luo, Rui Wang.

**Writing – original draft:** Xu Luo.

**Writing – review & editing:** Xin Zhang, Xin Wen, Wen Xie.
